# Steps initiated for sustainability of faculty mentorship program at Aga Khan University Medical College: A mixed method study

**DOI:** 10.12669/pjms.41.3.10856

**Published:** 2025-03

**Authors:** Rehana Rehman, Fauzia Khan, Tazeen Saeed Ali, Rahila Ali

**Affiliations:** 1 Rehana Rehman Professor, Department of Biological and Biomedical Sciences, Aga Khan University, Karachi, Pakistan; 2 Fauzia Khan Professor, Department of Anaesthesiology, Aga Khan University, Karachi, Pakistan; 3 Tazeen Saeed Ali Professor & Associate Dean, School of Nursing & Midwifery, Aga Khan University, Karachi, Pakistan; 4 Rahila Ali Assistant Professor, Department for Educational Development, Aga Khan University, Karachi, Pakistan

**Keywords:** Faculty mentorship, Mentoring, Sustainability

## Abstract

**Objective:**

To investigate and address challenges in faculty mentorship for sustainability of Faculty Mentorship Program (FMP) at Aga Khan University Medical College (AKU-MC)

**Methods:**

This is a mixed method study conducted from June 2021 to December 2023 at AKU-MC. It comprises of the exploration of qualitative insights into the challenges faced by mentors, mentees, and administrators, also incorporating quantitative measures of success to evaluate the sustainability of the program over time. Data was retrieved from four Focus group discussions (FGD) with mentors and mentees, eight in depth interviews with the administrators of FMP and leadership. Challenges of faculty mentorship were further identified by a panel discussion and briefing sessions with faculty members of different departments at AKU-MC. Challenges identified by three data sets were presented to the FMP committee, discussed in forum meetings and recommendations were proposed for the sustainability of the program.

**Results:**

During the interviews, mentors revealed ‘Limited Training and ‘Lack of Recognition’ as key challenges whereas mentees mentioned deficient skills in planning developmental goals. Administrators mentioned the need to enhance the visibility of the program as well as to update the resources. Panel discussion highlighted the need for recognition of mentors and providing them with protected time. Departmental meetings endorsed the need for sequential training of both mentors and mentees. To address these challenges educational grand rounds, international conferences and webinar were conducted to enhance the visibility of the program. Mentors were acknowledged by appreciation emails and tokens of appreciation. A series of workshops to enhance mentoring skills were planned and executed.

**Conclusion:**

The FMP at AKU-MC has devised a stepwise strategy to address challenges in faculty mentorship. This approach emphasizes collaboration with departments and the active involvement of leadership. By incorporating rewards, recognition, and a series of workshops, the program aims to foster a strong mentorship culture within the university. These efforts align with the objective of investigating and resolving mentorship challenges to ensure the sustainability of the Faculty Mentorship Program at AKU-MC.

## INTRODUCTION

Mentoring is cantered to support the professional and personal development of mentees. Mentors provide career counselling to help mentees during the stressful period of role inductance.^1^ Comprehensible, high-grade, and operational mentoring is considered to be a strategy for the development of the faculty.^2^ Faculty mentorship programs provide support, guidance, and professional development prospects for faculty members, prevent burnout as well as add to the prestige of an institution.^3^-^5^ These programs can be effective only when they consider the needs of mentors as well as mentees.^6^ There is a dire need to train mentors to understand their boundaries, roles and responsibilities as well as recognize the distinct needs of mentees.^7^

The Faculty Mentorship Program (FMP) at Aga Khan University Medical College (AKU-MC) was initiated in 2019 and continued with the faculty development activities.^8^ The broad goal is: ‘to create a conducive learning environment for professional development of faculty’. The goals are “to provide an opportunity to all faculty members with a career mentor, especially the junior and new faculty, support and facilitate faculty professional development through mentor /mentee relationship, provide a strong central structure, resources and leadership to support faculty mentoring at AKU, develop a comprehensive mentoring curriculum to enhance mentor/mentee competencies at AKU and build a mentoring database including processes and outcomes to support and evaluate mentoring activities”.

An exploration of the program recognized challenges faced by mentors, mentees and administration in the program.^8^ A workshop organized by the Department for Educational Development at AKU-MC highlighted the need to take the opinions of faculty members regarding challenges and way forward in mentoring^4^. Literature supports that mentorship experiences should be tailored to mentees’ need to avail maximum benefits of the relationship.^9^ Effective communication, time management, and documentation of all the proceedings are an important aspect of a mutually beneficial mentor-mentee relationship.^10^ It is imperative to investigate the mentoring needs of mentees to be addressed.^11^ Therefore, the study was designed to investigate and address challenges in faculty mentorship and deliberate steps to overcome them for the sustainability of FMP at AKU-MC.

## METHODS

This is a mixed method study; in continuation of a qualitative exploratory study conducted from June 2021 to December 2023.The study allows for the exploration of qualitative insights into the challenges faced by mentors, mentees, and administrators, while also incorporating quantitative measures of success to evaluate the sustainability of the program over time.

### Ethical Approval:

The study was approved by the Ethical Review Board of Aga Khan University (2021-6127-17832, 2022-6127-21635, 2023-6127-25374).

All mentors and mentees in FMP who attended at least two mentor-mentee sessions were invited to take part in a Focus Group Discussion (FGD). For the in-depth interviews (IDIs) program administrators; chair, co-chair, and coordinators who had served for a minimum of one year, were selected. Additionally, the Dean and Associate Dean of AKU-MC were requested for the interviews. All interviews were conducted in AKU-MC. Data was collected from four mentors, five mentees, six program administrators, Dean and Associate Dean of AKU-MC. Participants were invited through email and written informed consent was acquired before all interview sessions. They were also given a copy of signed consent for their future reference. The guidelines or protocols followed during FGDs (e.g., semi-structured guide. After each interview, verbatim transcripts were prepared and subjected to member checking to ensure accuracy. Codes were then generated from the data, organized into categories, and subsequently grouped to form the main themes. Challenges were derived from the **themes** emerged from these data collection tools.

In addition to the challenges identified during FGDs and IDIs, a larger audience from AKU-MC was invited to participate in the flagship panel discussion event, “Mentorship: Experiences and Challenges” held on 22^nd^ August, 2022. This event welcomed all faculty members—mentors and mentees—engaged in faculty mentorship, regardless of whether they were formally part of the FMP. Invitations for this event were extended to a broader audience, ensuring inclusivity and a wide range of perspectives. The event had 20 attendees, representing various departments and roles within the institution. The session was moderated by chair of the forum who guided the discussion, ensured equal participation, and maintained focus on the study’s objectives. This event featured an international speaker, who discussed different perspectives of faculty mentorship. The following panel discussion comprised of two mentors and mentees who successfully completed their 3-year faculty mentorship journey. Mentors shared their success stories, mentees talked about their challenges and both responded to inquiries of faculty members about faculty mentorship. During the discussion, moderator asked a series of questions outlined in the annexure 1. The session was recorded.

As per suggestions of the panellists, representatives of FMP then conducted briefing sessions with the departments of Obstetrics & Gynecology and Pathology & Laboratory Medicine from ‘May till August 2023’ to further determine the challenges. All the identified challenges from three data sets (interviews, panel discussion and departmental meetings) were presented to the FMP committee, discussed in program meetings and recommendations were proposed for the sustainability of the program.

## RESULTS

**Fig.1:**
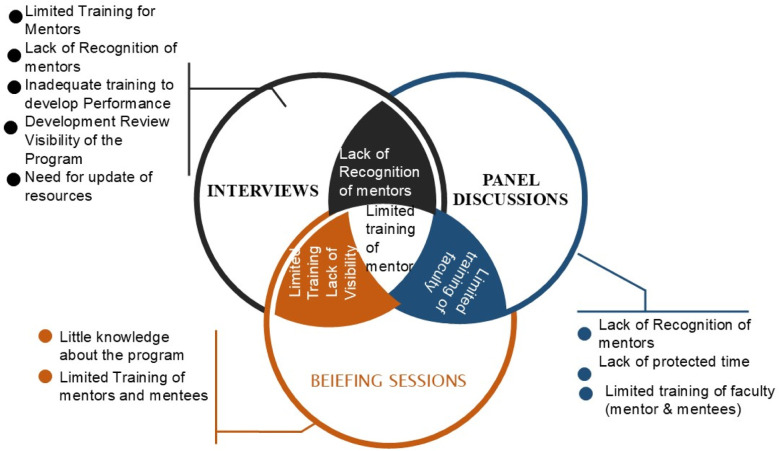
Challenges of faculty mentorship.

During the interviews, important challenges for mentors (Table-I) were ‘Limited Training’ and ‘Lack of Recognition’ whereas mentees’ deficient skills in planning developmental goals were mentioned by the mentees. Administrators declared the need to enhance the visibility of the program and an attempt to update the resources. The panel discussion highlighted the need to give recognition to mentors and provide them with protected time. Departmental meetings endorsed the need for sequential training of both mentors and mentees. In order to mitigate the challenges, suggestions from the study participants where all data sets were organized and common challenges listed (Fig.1). Lack of visibility of FMP was identified by administrators and during discussions with the department. Limited training of mentors was a common challenge identified during interviews and departmental meetings, whereas lack of recognition. Limited training of mentors was a common challenge from all data sets.

**Table-I:** Challenges identified by Interviews, Panel Discussion and Departmental Meetings with suggestions for improvement

Challenges	I) Excerpts from interviews (verbatim and descriptive)	Suggestions for improvement
***Mentors***		
Limited Training for Mentors	“*Not all faculty members have been trained in mentorship techniques*”	Providing mentor training can help mentors develop the skills they need to effectively support their mentees.
Lack of Recognition	“*In spite of spending time and energy, there was no recognition*”	By the department or by the institute no awards, incentives or encouragement was provide
***Mentees***		
Inadequate training to develop Performance & Development Review	Skills to plan goals were inadequate	Goal setting and structured guideline with periodic evaluation of milestones should be communicated through workshops provided
***Administrators: Mentoring Programs***		
Visibility of the Program	Faculty members do not know about the procedures to get enrolled in the program	Enhance visibility of the program
Need for update of resources	Administrative support was pointed out to be deficient	
Challenges	II) Reflections from Panel Discussions	Suggestions for improvement
Lack of Recognition of mentors Lack of protected time Limited training of faculty	Senior faculty members highlighted the importance of recognition of mentors during appraisals and at the time of promotions. Mentors demanded protected time for activity	Mentors should be recognized. The time spent in mentorship should be given credit during appraisal
Challenges	III) Reflections from Departmental Meetings	Suggestions for improvement
Little knowledge about the program	It was observed that junior faculty members did not know about the process of getting enrolled in the program	The program’s awareness should be increased.
Limited training of mentors and mentees	There is a need of sequential trainings of both mentors and mentees in exclusive workshops for both	Departments should nominate faculty members who should attend the workshops

All the initiatives taken by FMP to increase the visibility within and outside the university by participation in educational grand rounds, international conferences and training of faculty through workshops are illustrated in Table-II. In addition, mentors were acknowledged with appreciation letters from the Dean and received tokens of appreciation during events. Series of workshops to enhance mentoring skills were planned and executed (Table-II). Participants of workshops expressed commitment to several improvements in their professional practices like orientation to Professional Development Review, apply framework for goal setting and progress tracking and work on personal barriers and set realistic, measurable, and time-bound goals, create ‘Mentorship portfolio’ and use tools provided in the workshop for effective mentorship, conduct SWOT analysis for better mentoring and apply mentorship concepts to clinical and leadership roles.

**Table-II:** Steps taken by Faculty Mentorship Program at AKU-MC to mitigate the challenges.

Challenge	Purpose	Activities
Limited visibility of the Program	To increase awareness and visibility of the program within AKU	1.Education Grand Round (EGR): "Faculty Mentorship: Avenues, Challenges, and Prospects"
		2.Plenary Talk at PPS-18 Conference: "Faculty Mentorship: Navigating Pathways, Confronting Complexities, and Envisioning Future Prospects"
		3.Talk for PMA 93rd Webinar: "Faculty Mentorship in Universities: Empowerment for Excellence and Impact"
Limited training of faculty members	To help faculty members define goals for personal and professional development, and understand the value of being both a mentor and a mentee	A. Mentorship Workshop: "Faculty Mentoring: A Way Towards Synergistic Mentoring
		B. Workshop: "Mentorship; Building Stronger by Digging Deeper
		C. Being a Mentee; Empowering Journey of Personal & Professional Growth
Lack of recognition of mentors	To acknowledge the contributions of mentors within the program	1. Recognition for FMP Members: Souvenirs were presented to members of the Faculty Mentorship Committee.
		2. Recognition for Mentors: Souvenirs were distributed to mentors involved in the program.

## DISCUSSION

The research highlights initiatives taken by FMF to identify areas of improvement with the efforts to inculcate a culture of mentorship. It was observed that junior faculty members did not know about the process of being enrolled in the program which highlighted the need to enhance visibility of the program. Mentors highlighted the need of training of mentors. Recognition for mentorship can increase self-esteem, readiness to participate in mentorship, promote career progression, satisfaction and retention of mentors.^12^ Data collected from exploratory study and panel discussion organized by FMP highlighted the importance of recognition of mentors. Similar recognition of excellence by introducing additional new awards by the Executive Board of the Society for Music Perception and Cognition (SMPC) has also been observed.^13^

**APPENDIX-1:** 

1. What barriers have you encountered in fostering a productive mentor-mentee relationship,
2. How did you address them?
3. Have you encountered situations where a mentorship relationship did not meet expectations?
4. How did you navigate those situations?
5. How do you balance mentorship responsibilities with other professional obligations?
6. Can you mention any challenges?
7. What role do you think leadership and departmental support play in overcoming mentorship challenges?
8. Are there any cultural or institutional barriers that affect mentorship at AKU-MC?
9. How can these be addressed?
10. How do you handle conflicts or differences of opinion with your mentee?

A number of strategies can hence be employed for recognition of the mentors like support in their career progression, promotions, rejoinders in meetings, rewards and remunerations.^14^ Considering the importance of recognition of mentors on national and international levels, leadership at AKU-MC organized an event to recognize members of FMP by giving souvenirs to mentors. In addition to that, a letter of appreciation was sent to all the mentors in the program. A mentorship award was also announced from 2021 to date with the same objective.

Quality mentoring helps mentees to flourish in their career paths, increases the likelihood of securing publications and grants with greater chances of being promoted and recognized in their respective fields.^15^ An important fact highlighted in the study was the need for training mentors so that mentors should not train mentees the way they have been trained.^14^,^15^ The Push-Pull Mentoring Model also suggested comprehensive training workshops, sessions, and courses to prepare mentees with the mandatory expertise and wisdom to serve as mentors.^16^ These programs should emphasize active listening, empathy, goal setting, and providing constructive feedback to both mentors and mentees.^16^ Therefore, FMP designed a series of workshops to train them formally so that they know the essence of mentorship and help the juniors in their professional development plans.^17^

Mentees in our study emphasized the importance and need of goal setting. This corroborates with the study emphasizing the need to teach SMART goal-setting skills to the students.^18^ The administrators interviewed in the program identified the need for strong administrative support which has been documented in the literature.^3^ To address these issues, the program needs to prioritize mentor training, increase visibility, and provide mentors with adequate recognition and support. Additionally, efforts should be made to standardize mentorship programs across regions/countries through collaboration between institutions/programs within each field which is the future destination of the program.

### Limitations:

The data collected through interviews and surveys is subject to self-reporting bias, as participants sometimes provide incomplete information. The study has focused on a specific type of mentoring program, such as faculty mentoring, which may limit the transferability of the findings to other types of mentoring programs. Although mentors were acknowledged through appreciation emails and recognized during special events, no specific initiatives were implemented exclusively for them. The data from interviews, panel discussions, and briefing sessions was collected by the principal author, which introduces the possibility of research bias.

## CONCLUSION

The FMP at AKU-MC has devised a stepwise strategy to address challenges in faculty mentorship. This approach emphasized collaboration with departments and the active involvement of leadership. By incorporating rewards, recognition, and a series of workshops, the program aims to foster a strong mentorship culture within the university. These efforts align with the objective of investigating and resolving mentorship challenges to ensure the sustainability of the Faculty Mentorship Program at AKU-MC.

### Authors’ Contribution:

**RR:** Designed and executed the study.

**RA** and **TSA:** Supervised the whole project from data collection to data analysis.

**FK:** Analyzed challenges faced by mentors and mentees of the FMP and proposed steps to overcome the challenges.

**RA:** Responsible for the accuracy of study.

All authors took part in the write-up of the manuscript, read and revised the content.

### Recommendations:

FMP plans to initiate follow-up studies to assess the long-term impact of the proposed initiatives on the sustainability of the program. In addition to that forum will establish a framework for continuous mentorship training and recognition to ensure the program’s scalability and sustainability.
